# Photocatalytic degradation of sulfamethazine in aqueous solution using ZnO with different morphologies

**DOI:** 10.1098/rsos.171457

**Published:** 2018-04-11

**Authors:** Zhigang Yi, Juan Wang, Tao Jiang, Qiong Tang, Ying Cheng

**Affiliations:** 1 College of Chemistry, Leshan Normal University, Leshan, Sichuan 614004, China; 2 Environmental Monitoring Station of Environmental Protection Bureau of Rizhao Lanshan, Lanshan, Shandong 276800, China

**Keywords:** sulfamethazine, photocatalytic degradation, decomposition kinetics, ZnO

## Abstract

In this study, photocatalytic experiments of 20 mg l^−1^ sulfamethazine (SMN) in aqueous solution containing ZnO with different morphologies, tetra-needle-like ZnO (T-ZnO), flower-like ZnO (F-ZnO) and nanoparticles ZnO (P-ZnO), were performed. The results indicated that photocatalytic degradation of SMN was effective and followed the pseudo-first-order reaction, but the degree of SMN mineralization showed obvious differences using ZnO with different shapes. After 12 h irradiation, 86%, 71% and 50% of the initial total organic carbon was eliminated in SMN suspension containing T-ZnO, F-ZnO and P-ZnO, respectively. The release ratio of sulfur was close to 100% in the presence of T-ZnO, but reached to 86% and 67% in the presence of F-ZnO and P-ZnO, respectively. The release ratio of nitrogen was about 76%, 63% and 40% using T-ZnO, F-ZnO and P-ZnO as photocatalyst, respectively. The morphology of ZnO played an important role in determining its catalytic activity. Seven intermediates were observed and identified in the UV/T-ZnO reaction system by LC-MS/MS analysis, and a possible degradation pathway was proposed.

## 1. Introduction

To satisfy the growing demand of humans for animal protein, antibiotics have been used not only to treat disease in animal husbandry, but also to promote animal growth as feed additives in livestock and aquaculture [1–3]. In 2010, at least 63 200 tons of antibiotics were consumed by stock farming around the world [4]. By 2030, the consumption of antibiotics is projected to rise by two-thirds, to 105 600 tons [5]. The usage of antibiotics produces residues in its parent form without absorption and metabolism by animals, which are directly or indirectly introduced into the aquatic and terrestrial environments [2,3]. Although the concentration of residues detected in the environment is quite low (ng l^−1^–mg l^−1^), the ecotoxicity of residues at mg l^−1^ levels has been reported [6]. Many research studies indicated that the antibiotic residues were resistant to conventional chemical and biological treatment methods [7]. The accumulation of antibiotic residues in the environment might make the antibiotic ineffective in diseases treatment, causing a serious public health issue on account of the development of antibiotic-resistant bacteria [8,9]. Effective ways to eliminate the discharged antibiotic residues are required for environmentally sustainable development [10,11].

As a promising method, photocatalytic degradation in the presence of semiconducting materials has exhibited high efficiency in removing a great variety of organic compounds [12]. Among various semiconducting materials, most attention has been paid to TiO_2_ because of its high photocatalytic activity, resistance to photo-corrosion, biological immunity and low cost. For example, Baran *et al*. reported the photocatalytic oxidation of sulfonamides with TiO_2_ and TiO_2_–FeCl_3_ systems [13]. Elmolla *et al*. used UV-A/ZnO and UV-A/TiO_2_ photocatalytic systems to treat a synthetic antibiotic formulation wastewater containing amoxicillin, cloxacillin and ampicillin at a total concentration of about 300 mg l^−1^ [14,15]. TiO_2_ is the most common photocatalyst in the field of photocatalytic research and application. But in fact, ZnO shows excellent photocatalytic performance and is harmless, stable, cheap etc. So far, many studies have confirmed that ZnO exhibits more efficiency than TiO_2_ in photocatalytic degradation of organic pollutants [16]. However, like other semiconductor materials, ZnO also has the main disadvantage that the recombination of $h_{VB}^{+}-e_{CB}^{-}$ results in negative effects on photocatalytic activity. In order to overcome this shortcoming, various efforts have been made. ZnO/Ag/CdO [17], FexZn1_xO [18] and ZnO/CeO_2_ [19] and other nanocomposites were synthesized through easy control methods. After the modification of ZnO, the photocatalytic performance has been obviously improved. As a matrix material, ZnO with different structures and morphologies has been fabricated in different ways [20–23]. The morphology of nanomaterials played a key role in determining the catalytic activities. Pandiyarajan *et al*. [24] synthesized CuO with different morphologies, and concluded that the smaller size and high surface area of the spherical nanostructures contribute to higher catalytic properties. Gnanasekaran *et al*. [25] found that the superior photocatalytic activity of ZnO was due to its spherical shape and crystallinity. Saravanan *et al*. [26,27] prepared ZnO in different shapes and sizes using different methods, and identified the relationship between shape and photocatalytic activity. Although many studies have been carried out on the relationship between photocatalytic activity and crystallinity, surface area, morphology of semiconductor nanoparticles in detail, there were few studies on the photocatalytic behaviours of antibiotics using ZnO with different morphologies. The studies on these are of great significance to promote the development of photocatalytic technology in the field of antibiotic wastewater treatment.

Sulfamethazine (SMN) was widely used to control diseases and promote animal growth in livestock production [28,29]. Meanwhile, SMN was commonly detected in natural water or secondary effluent [30]. In this study, SMN was chosen as the representative substance of antibiotics, ZnO particles with three different shapes were used as photocatalysts, to study the photocatalytic degradation behaviours of antibiotics in aqueous solution and the influence of different shapes of ZnO on its photocatalytic activity.

## 2. Material and experimental methods

### 2.1. Materials

SMN with high purity standards (99%) was purchased from Aladdin Industrial Corporation (Shanghai, China). Acetonitrile (HPLC grade) and other chemical reagents (analytical grade) were bought from Best Reagent (Chengdu, China). Ultrapure water was used to prepare SMN solutions and HPLC eluent.

### 2.2. Preparation and characterization of ZnO

Three different morphologies of ZnO used in this study were prepared by different methods. Tetra-needle-like ZnO (T-ZnO) came from Key Laboratory of Advanced Technologies of Materials of Ministry of Education, Southwest Jiaotong University; the preparation method of T-ZnO was described in an earlier report [31]. ZnO nanoparticles (P-ZnO) were synthesized through a low-temperature co-precipitation process in an aqueous solution. During synthesis, the concentration of Zn(CH_3_COO)_2_·2H_2_O solution was 0.1M, the NaOH aqueous solution was added dropwise to the Zn(CH_3_COO)_2_·2H_2_O solution as the basic ratio (b = nOH^−^/nZn^2+^ = 8) and the mixture was vigorously stirred for 2 h. The reaction system was set at 60°C [23]. The flower-like ZnO (F-ZnO) was prepared using Zn(NO_3_)_2_·6H_2_O and NH_3_·H_2_O as precursor materials by an ultrasonic-assisted solution method. A certain amount of Zn(NO_3_)_2_·6H_2_O was dissolved in deionized water. An aqueous solution NH_3_·H_2_O was added dropwise to Zn(NO_3_)_2_·6H_2_O solution until the pH of reaction system reached about 12. Whereafter, the mixed solutions were ultrasonically oscillated at 25°C for 5 h. At the end of the processes, P-ZnO and F-ZnO particles were separately synthesized, filtered and washed with methanol and deionized water several times and subsequently calcined for 2 h under 300°C [22]. The morphology of ZnO was observed with a scanning electron microscope (Quanta 200, Fei, Holand) operating at 20.0 kV. The crystal structure of ZnO particles was characterized by an X-ray diffractometer (DX-2500, Dandong fangyuan instrument Co., Ltd, China) with Cu K*α* radiation (40 kV, 40 mA, *λ* = 0.15418 nm). The UV–visible diffuse reflectance spectroscopy (uv-DRS) of ZnO samples was detected by UV–visible spectrophotometer (TU-1901, Persee, China). The specific surface area of the ZnO samples was measured on a specific surface area analyser (TriStar 3000 Analyzer, America).

### 2.3. Photocatalytic experimental set-up

**Figure 1.**
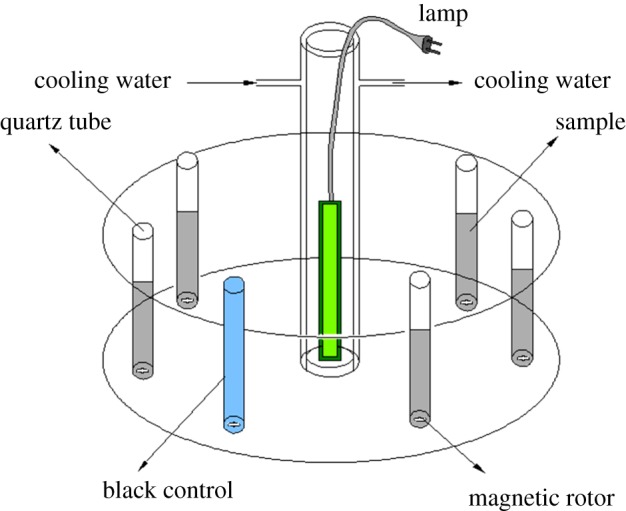
Diagram of the experimental set-up.

A merry-go-round photochemical reactor with magnetic stirring was employed (illustrated in figure 1). The high-pressure Hg lamp (300 W), emitting predominantly at 365 nm UV light, was put in a quartz sleeve and located at the centre of the reactor. The SMN solutions (20 mg l^−1^) containing different morphologies of ZnO particles (2 g l^−1^) were put in 50 ml quartz tubes and equidistantly placed around the Hg lamp. The suspensions were stirred constantly for 30 min in the dark to ensure equilibration of adsorption/desorption of SMN onto the ZnO surface, then the 300 W high-pressure Hg lamp was turned on. During photocatalytic reaction, the reaction system was set at 25°C using a temperature control unit. The pH of suspensions was adjusted to 7, the neutral form of SMN dominates (greater than 80%) under this condition [32]. The samples were taken at scheduled time intervals and separated using centrifugation (3000 r.p.m., 5 min), then supernatants were filtered with a membrane syringe filter (pore size: 0.2 µm) and sent for analysis. All of the experiments were performed in triplicate and average values were quoted as results.

### 2.4. Analytical methods

The concentration of SMN at different irradiation times was determined by HPLC (LC-2010HT, Shimadzu) coupled with UV--vis detection (LC-UV--vis). A C_18_ column (2.1 mm × 100 mm). The detection wavelength of SMN was recorded at 268 nm. The eluents were (A) H_2_O + 0.1% formic acid and (B) acetonitrile at 80 : 20 ratio and the flow rate was set to 0.2 ml min^−1^.

The structural identification of photoproducts was carried out with a Shimadzu LC-20A liquid chromatograph system coupled with Shimadzu LCMS-8030 triple quadrupole mass spectrometer (LC-MS/MS). The eluent and column used for the separation of the parent compound and its photoproducts were the same as those of LC-UV--vis analyses, but the ratio of A and B of eluent was changed during the run: started with 10% of B, then it rose to 60% after 10 min and then to 90% in 8 min, the content of B dropped to 10% in 10 min and remained the ratio until the end of the run. The detection was performed with an electrospray ionization (ESI) source. The following conditions were set: capillary voltage 4000 V, drying gas temperature 300°C, drying gas flow 12 ml min^−1^, nebulization gas 35 psi. Fragmentor voltages were adjusted between 10 and 30 V to obtain precursor ions of degradation products.

Total organic carbon (TOC) was detected by TOC analyser (TOC4100, Shimadu). The concentrations of ${\text{SO}}_{4}^{2-}$, ${\text{NO}}_{3}^{-}$ and ${\text{NH}}_{4}^{+}$ during SMN photocatalytic reaction were measured using ion chromatography (ICS-90; Dionex).

## 3. Results and discussion

### 3.1. Morphology, structure and optical properties of ZnO

**Figure 2.**
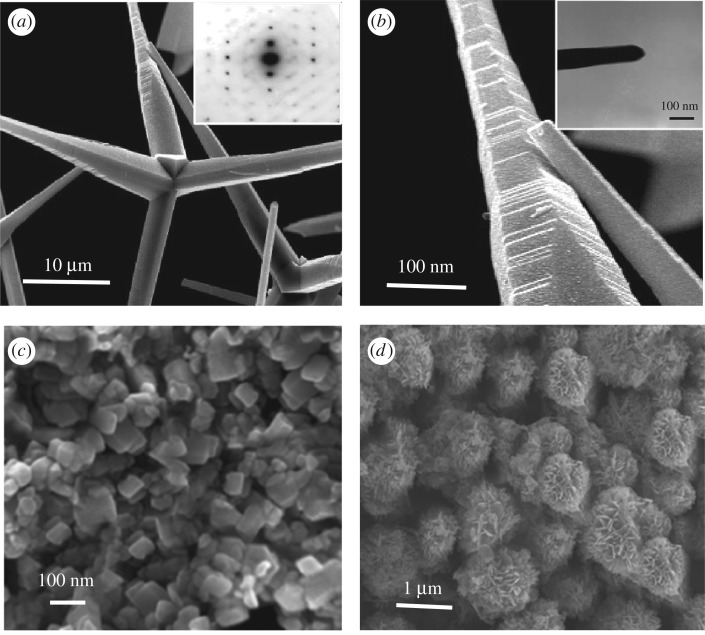
SEM image of ZnO samples: (*a*) SEM image of T-ZnO, the inset is the electron diffraction pattern of T-ZnO, (*b*) enlargement image of the surface of T-ZnO, the inset is TEM image of apex of needle, (*c*) SEM image of P-ZnO, (*d*) SEM image of F-ZnO.

The SEM images of the three kinds of ZnO samples are demonstrated in figure 2. Significant differences in the morphology and size were observed on account of the different preparation methods. As shown in figure 2*a*,*b*, each crystalline body of T-ZnO had four needle-like legs extending from a core part. The size of the basal diameter and the apex of each needle were about 1–3 µm and 50 nm, respectively. The length of each needle was 10–30 µm, and there were many obvious growth steps and edge structures on the acicular parts of T-ZnO. The electron diffraction pattern (inset in figure 2*a*) displayed that the needle was of a single crystal. The morphologies of P-ZnO and F-ZnO are demonstrated in figure 2*c*,*d*, respectively. Most of P-ZnO appeared hexagonal and the average particle size was about 80 nm in length and 40 nm in diameter. The detailed feature of F-ZnO was flower-like microstructure with diameter in the range of 1–2 µm, which was composed of nanoplates.

**Figure 3.**
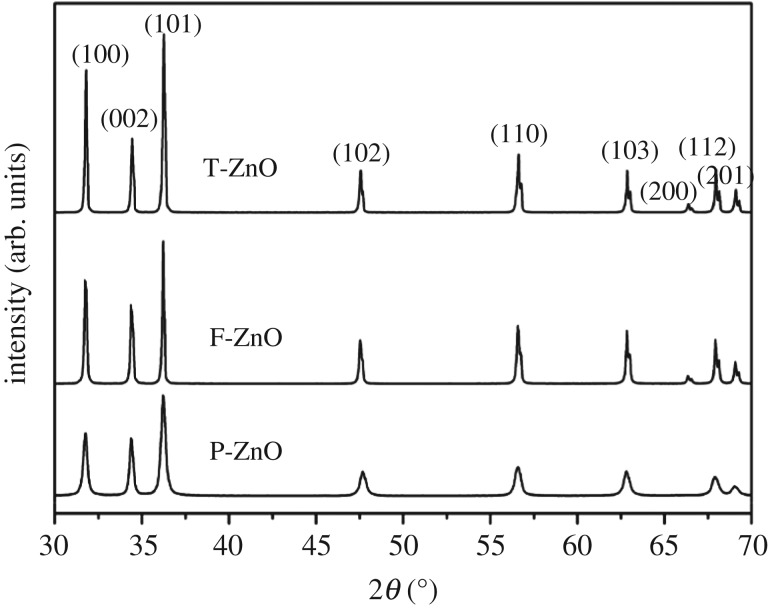
XRD patterns of the ZnO samples.

The XRD patterns of ZnO particles are shown in figure 3. The observed diffraction peaks of T-ZnO, F-ZnO and P-ZnO corresponded to crystallized ZnO with hexagonal wurtzite structure according to the diffraction data (JCPDS no. 36-1451) [33]. No additional characteristic peaks from impurities were detected. The structural information and the average crystallite size estimated from the FWHM of (100), (002) and (101) reflections of T-ZnO, F-ZnO and P-ZnO are listed respectively in table 1. The specific surface area of the three ZnO samples was also measured and listed in table 1.

**Table 1.** The structural information, crystal size and bandgap of ZnO samples.

			lattice parameters		
samples	specific surface area (m^2^ g^−1^)	crystal size (nm)	a (Å)	c (Å)	E_g_ (eV)
T-ZnO	0.29	104	3.2504	5.2068	3.253
F-ZnO	2.43	84	3.2502	5.2065	3.252
P-ZnO	5.74	34	3.2526	5.2106	3.251

**Figure 4.**
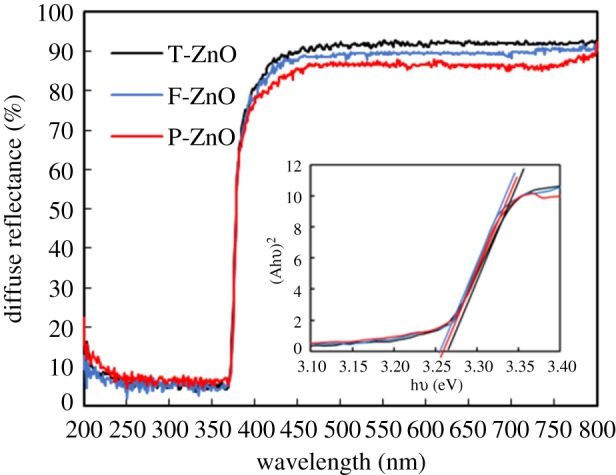
UV–vis diffuse reflectance spectra of ZnO particles, the inset is plots of (Ahυ)^2^ versus hυ representing the estimation of direct band gap of ZnO samples.

The UV–vis diffuse reflectance spectra of T-ZnO, F-ZnO and P-ZnO are displayed in figure 4. The optical band gap E_g_ of ZnO samples estimated by the extrapolation of the linear portion of the plots of (Ahυ)^2^ versus hυ is listed in table 1 [34]. All ZnO particles exhibited a strong, similar absorption at wavelengths in the range 200–400 nm. It was consistent with the values reported for ZnO nanoparticles [35,36]. The E_g_ of the three ZnO samples was lower than known E_g_ of bulk ZnO (3.37 eV), which was caused by the existence of some point defects within ZnO crystal lattice [23].

### 3.2. Photocatalytic decomposition kinetics of sulfamethazine under different conditions

In order to evaluate the efficiency of ZnO for photocatalytic degradation of SMN, the experiments concerning the photocatalytic decomposition of 20 mg l^−1^ SMN solution containing 2 g l^−1^ ZnO with different shapes were performed. Meanwhile, the blank experiment for illuminated SMN without ZnO was carried out.

On account of the low concentration of SMN, the pseudo-first-order kinetic model (equation (3.1)) was applied to analyse the SMN photocatalytic decomposition [37].

**Figure 5.**
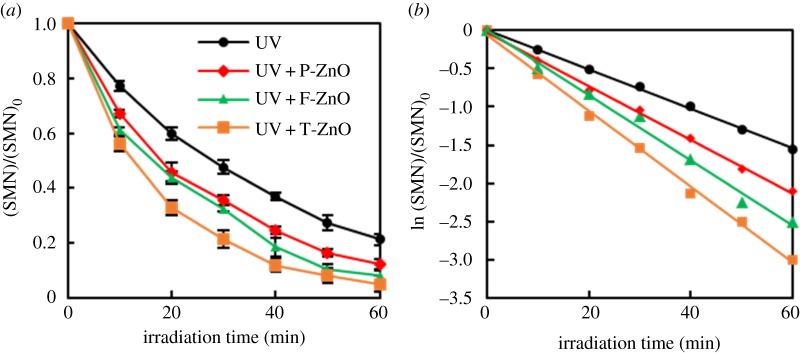
Degradation ratio (*a*) and kinetics (*b*) of SMN photocatalytic degradation by different ZnO samples.

3.1 $$-\ln\left(\frac{\left[\text{SMN}\right]}{{\left[\text{SMN}\right]}_{0}}\right)=kt,$$

where [SMN]_0_ and [SMN] were the initial concentration and the concentration of SMN at the reaction time *t*, respectively. The *k* (min^−1^) was the reaction rate constant. The results are displayed in figure 5*a*,*b*. As shown in figure 5*a*, the blank experiment indicated that the photolysis of SMN was obvious. The removal ratio of SMN by photolysis was 78% after 60 min irradiation and the rate constant *k* of SMN was 2.58 × 10^−2^ min^−1^ without ZnO. In the presence of T-ZnO, the removal ratio and rate constants increased significantly, i.e. 95% and 4.95 × 10^−2^ min^−1^, respectively. The increasing extent of three different ZnOs decreased in the following order: T-ZnO, F-ZnO, P-ZnO. The results indicated that the process of photolysis and photocatalytic degradation by ZnO of SMN followed the pseudo-first-order reaction, the presence of ZnO was beneficial to the degradation of SMN, and the photocatalytic activity of T-ZnO was best.

### 3.3. Degree of sulfamethazine mineralization under different conditions

As mentioned in many research studies, most of the organic carbons were still retained although the photolysis of antibiotics under UV was effective. The studies on antibacterial activity and toxicity of the intermediates produced by photolysis of sulfonamides [32,38], fluoroquinolone [39,40], tetracycline [41,42] and others indicated that the mixed by-products showed an increasing toxicity, and the solutions after photolysis still had certain residual antibacterial activity. Furthermore, the complete decomposition of organic contaminant to inorganic molecules is the main purpose in wastewater treatment. Under various experimental conditions, the destruction of organic compounds is different. Based on these, the efficiency of ZnO samples with different morphologies for SMN mineralization was evaluated in this study.

The overall reaction of SMN mineralization is presented as equation (3.2):
3.2
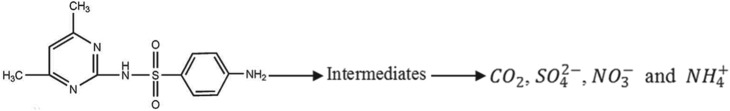


**Figure 6.**
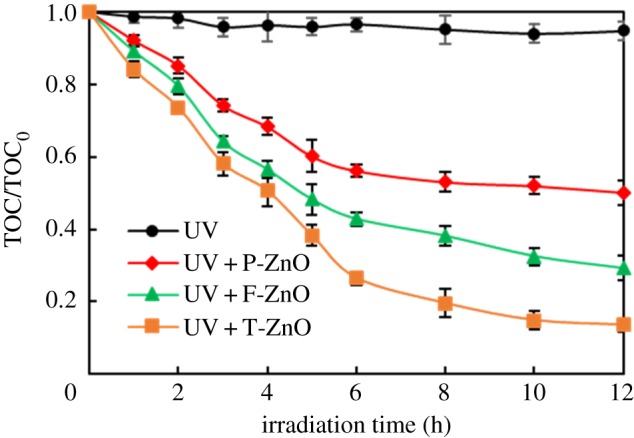
Removal ratio of TOC during SMN photocatalytic degradation by different ZnO samples.

In this study, the efficiency of ZnO samples with different morphologies for SMN mineralization was evaluated by monitoring the total organic carbon (TOC) during the photocatalytic degradation of SMN. The changes in TOC content measured during the SMN photocatalytic decomposition are shown in figure 6. After 12 h irradiation, no significant removal of TOC was observed in the absence of ZnO, the addition of ZnO resulted in significant decrease in TOC content, but the decline level was different due to the morphological change in ZnO. During the first 12 h, 86% of the initial TOC in SMN suspension containing T-ZnO was eliminated; meanwhile, 71% and 50% was obtained in the presence of F-ZnO and P-ZnO, respectively.

**Figure 7.**
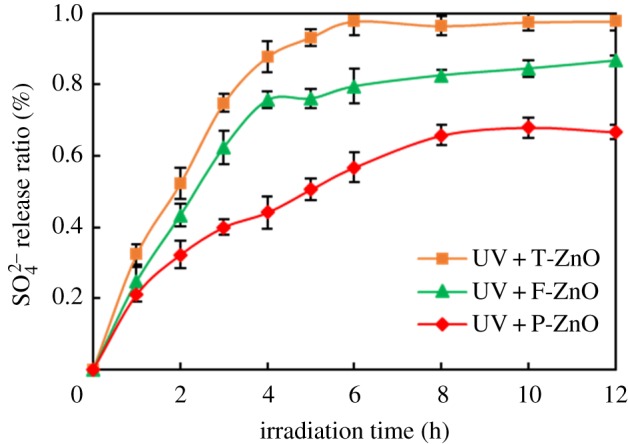
${\text{SO}}_{4}^{2-}$ release ratio during SMN photocatalytic degradation by different ZnO samples.

**Figure 8.**
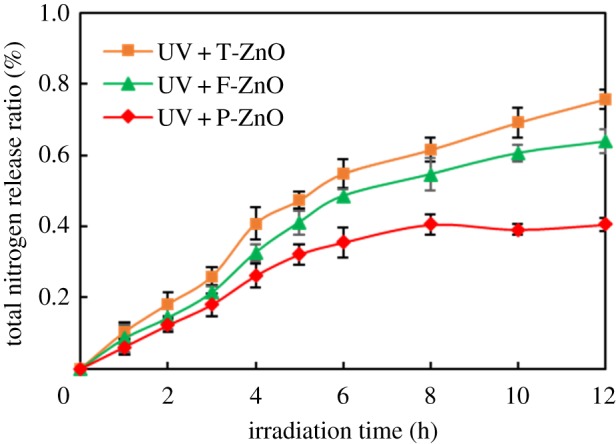
Total nitrogen release ratio during SMN photocatalytic degradation by different ZnO samples.

The mineralization degree of SMN can be further confirmed using release ratio of sulfur and nitrogen during the photocatalytic degradation of SMN. To suppose that SMN could be completely mineralized in the photocatalytic process, the maximum concentrations of inorganic sulfur and nitrogen released from SMN (20 mg l^−1^ initial concentration) would reach 2.29 mg l^−1^ and 4.01 mg l^−1^, respectively. The release ratios are shown in figures 7 and 8, which were calculated by testing the concentration of ${\text{SO}}_{4}^{2-}$, ${\text{NO}}_{3}^{-}$, ${\text{NH}}_{4}^{+}$ in the reaction system. The release ratio of sulfur was an efficient process, close to 100% in 6 h irradiation in the presence of T-ZnO, but reached to 86% and 67% after 12 h reaction in the presence of F-ZnO and P-ZnO. On the other hand, the organic nitrogen conversion to inorganic ions (${\text{NO}}_{3}^{-}$, ${\text{NH}}_{4}^{+}$) was an inefficient process under the same experimental conditions. The release ratio of nitrogen was about 76%, 63% and 40% using T-ZnO, F-ZnO and P-ZnO as photocatalyst, respectively.

Combining the results of decomposition kinetics of SMN (§3.2), as it could be seen, SMN could be converted to intermediates under irradiation of high-pressure mercury lamp, but the photolytic by-products were rarely broken down further. After the addition of ZnO as a photocatalyst, even though the intermediate products stayed for a certain amount of time, most of them were mineralized into inorganic molecules or ions after 12 h reaction. The photocatalytic efficiency of different ZnOs for SMN mineralization decreased in the following order: T-ZnO, F-ZnO, P-ZnO.

There were two main reasons for the difference in photocatalytic activity of different shapes of ZnO. On the one hand, the needle tip of T-ZnO grows along the [0001] direction, so resulting in the exposure of most of the $\left\{10\bar{1}0\right\}$ [43,44]. Related research showed that the photocatalytic activity of the ZnO increased with the peak intensity ratio of $\left(10\bar{1}0\right)$ to (0002) [45]. Xu *et al*. compared the activity for the production of three different ZnOs and found the oxygen vacancies in skin layers of the T-ZnO crystal played an important role in the formation of H_2_O_2_ and had a positive effect on the production of ·OH [34], which was beneficial to photocatalytic activity. On the other hand, the agglomeration of nanomaterials reduces the photocatalytic activity. Even though the size of T-ZnO and F-ZnO are micron grade, the top T-ZnOs needle is nanoscale and F-ZnO consists of nanoplates, which make T-ZnO and F-ZnO have the advantage of nanomaterials and harder to reunite than nanomaterials. The lower photocatalytic activity of the P-ZnO is attributed to the aggregation of the nanoparticles.

### 3.4. Intermediate product analysis and photocatalytic degradation pathway of sulfamethazine

**Figure 9.**
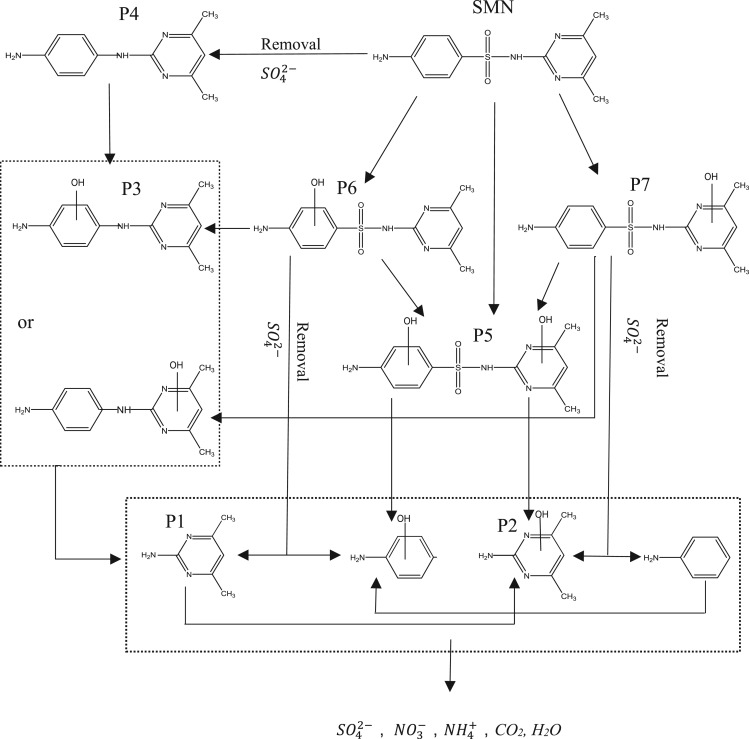
Major intermediate and proposed pathways of photocatalytic degradation of SMN under UV/T-ZnO.

In order to identify the intermediate products of SMN photocatalytic degradation, the solution obtained after SMN degradation for 30 min, using T-ZnO as photocatalyst, was purposely used to detect as many intermediates as possible. LC-MS/MS was employed to perform measurements. The total ion chromatogram (TIC) and the specific ion mass spectra of major intermediates are depicted in electronic supplementary material, figure S1. There were seven different peaks, excepting SMN. Based on the ion mass spectra of each peak, seven major intermediates, namely P1–P7, were identified in the UV/T-ZnO reaction system. P6 and P7 had the same molecular weight, *m/z* 295, 16 larger than that of SMN. Previous studies have reported that ·OH radicals formed during the photocatalytic process could attack the investigated molecule at any function group, resulting in a molecular weight increase of 16 [11,46,47]. Electronic supplementary material, figure S1 describes the different retention times and ion fragmentation of P6 and P7, which indicated that the hydroxyl group was added at the benzene ring and the dimethyl pyrimidine group of SMN, respectively. The intermediate with largest molecular weight was P5, 16 larger than that of P6 and P7. By comparing the specific ion mass spectra of P5, P6 and P7, we found P5 obtained from the hydroxylated reaction of the benzene ring and the dimethyl pyrimidine group. Research studies indicated that SO_2_ extrusion was a phenomenon that frequently occurs during sulfonamide degradation driven by UV photolysis. The intermediates were obtained by aminobenzene ring directly connecting to the pyrimidine ring, and then, the holes (h+) of photocatalyst attacking the carbon–nitrogen bond of the dinitrogen-substituted ring of the SMN would result in two by-products [48–50]. Based on these main reasons, P4 with *m/z* 21 564, less than that of SMN, was produced on account of SO_2_ removal. The hydroxylated reaction of P4 or the SO_2_ elimination from P5 and P6 could generate the by-product with *m/z* 231, named P3. P1 and P2 with smaller *m/z* were derived from the broken bond of the carbon–nitrogen bond by other intermediates. Combining the results of the previous section (§3.3), we could propose the possible photocatalytic decomposition pathway of SMN in the presence of UV/T-ZnO, which is demonstrated in figure 9. Hydroxylations on the benzoic ring and the pyrimidine ring of SMN, and SO_2_ extrusion on sulfonamide group via the direct cleavage of the S–N bond were suggested to be the major pathways of SMN photodegradation using ZnO under UV irradiation.

## 4. Conclusion

This study examined the kinetics of photolysis and photocatalytic degradation of SMN respectively in the absence and presence of ZnO with different morphologies, and the degree of SMN mineralization was evaluated by monitoring the total organic carbon (TOC), release ratio of sulfur and nitrogen during the photocatalytic degradation of SMN. The removal ratio of SMN (20 mg l^−1^) by photolysis was 78% after 60 min irradiation of high-pressure mercury lamp and the rate constant *k* of SMN was 2.58 × 10^−2^ min^−1^ without ZnO, but no significant removal of TOC was observed. In the presence of ZnO, the removal ratio and rate constants increased significantly and the increasing extent of three different ZnOs decreased in the following order: T-ZnO, F-ZnO, P-ZnO.

The degree of SMN mineralization decreased in the same order. There are three main reasons for the best photocatalytic activity of T-ZnO: (i) Growing along the [0001] direction of the needle tip of T-ZnO, results in most of the $\left\{10\bar{1}0\right\}$ being exposed. (ii) The oxygen vacancies in skin layers of the T-ZnO crystal have a positive effect on the production of ·OH, which is beneficial to photocatalytic activity. (iii) The micron size of T-ZnO makes T-ZnO harder to reunite, and the nanoscale top of needle makes it have the advantage of nanomaterials.

Seven intermediates were observed and identified in the UV/T-ZnO reaction system by LC-MS/MS analysis. Hydroxylations on the benzoic ring and the pyrimidine ring of SMN, and SO_2_ extrusion on sulfonamide group via the direct cleavage the S–N bond were suggested to be the major pathways of SMN photodegradation using ZnO under UV irradiation.

## Supplementary Material

Fig.S1

## Data accessibility

We have conducted our experiments systematically and reported their experimental procedure clearly in the experimental section and provided all necessary data in results and discussion section in the main manuscript.
