# Supplementary material for: Photocatalytic degradation of sulfamethazine in aqueous solution using ZnO with different morphologies
Source: R Soc Open Sci. 2018 Apr 11;5(4):171457. doi: 10.1098/rsos.171457 (PMC5936895; doi:10.1098/rsos.171457)
Supplement: Fig.S1 [file rsos171457supp1.docx]

Fig.S1 of Manuscript

Photocatalytic degradation of sulfamethazine in aqueous solution using ZnO with different morphologies

**Zhigang Yi****^[[1]](#footnote-1)^*, Juan Wang^[[2]](#footnote-2)^, Tao Jiang^a^, Qiong Tang^a^** **and Ying Cheng^a^**


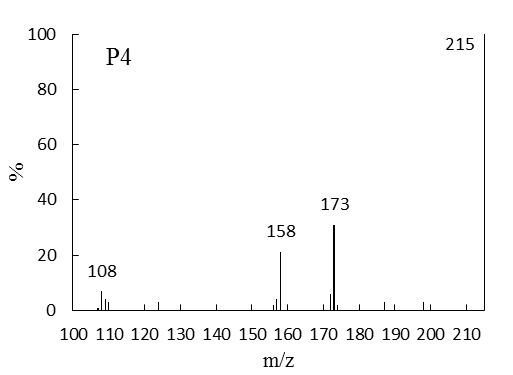

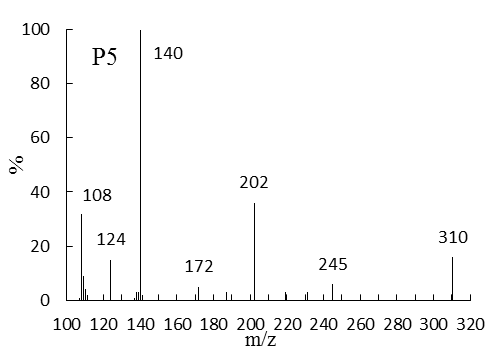

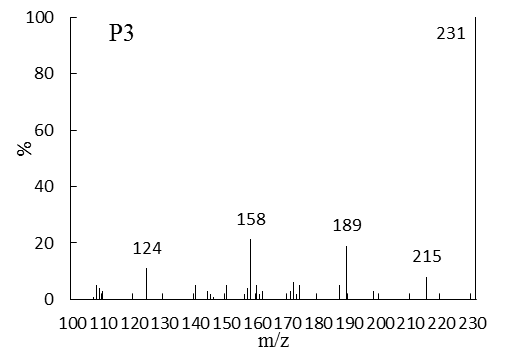

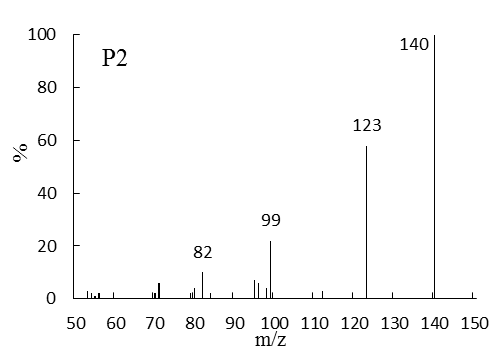

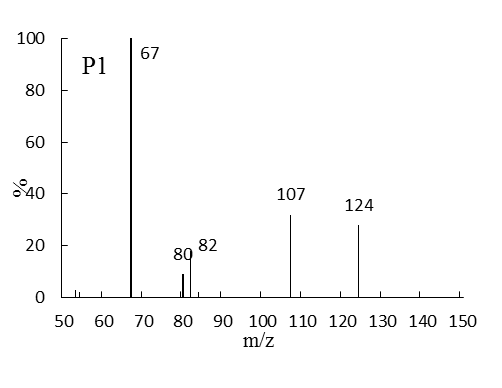

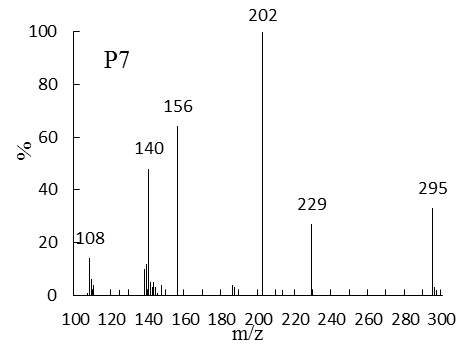

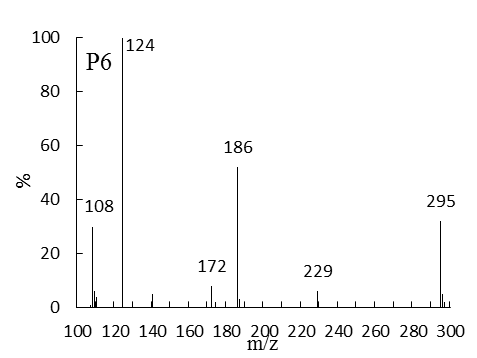


TIC

Fig.S1. Total ion chromatogram(TIC) and Major intermediates ion mass spectra(P1~P7)

of SMN

1. College of Chemistry, Leshan Normal University, Leshan, Sichuan 614004 China [↑](#footnote-ref-1)
2. Environmental Monitoring Station of Environmental Protection Bureau of Rizhao Lanshan, Lanshan, Shandong 276800, China

   *corresponding author: [yizhigang117@hotmail.com](mailto:yizhigang117@hotmail.com) [↑](#footnote-ref-2)
